# Ascorbate Uptake and Retention by Breast Cancer Cell Lines and the Intracellular Distribution of Sodium-Dependent Vitamin C Transporter 2

**DOI:** 10.3390/antiox12111929

**Published:** 2023-10-30

**Authors:** Citra Praditi, Stephanie M. Bozonet, Gabi U. Dachs, Margreet C. M. Vissers

**Affiliations:** 1 Mātai Hāora—Centre for Redox Biology and Medicine, Department of Pathology and Biomedical Science, University of Otago Christchurch, Christchurch 8011, New Zealand; citra.praditi@otago.ac.nz (C.P.); stephanie.bozonet@otago.ac.nz (S.M.B.); 2 Mackenzie Cancer Research Group, Department of Pathology and Biomedical Science, University of Otago Christchurch, Christchurch 8011, New Zealand; gabi.dachs@otago.ac.nz

**Keywords:** vitamin C transport, SVCT2, pharmacokinetics, subcellular compartments, MCF7, MDA-MB231, EO771, hypoxia

## Abstract

Ascorbate plays a vital role as a co-factor for a superfamily of enzymes, the 2-oxoglutarate dependent dioxygenases (2-OGDDs), which govern numerous pathways in cancer progression, including the hypoxic response and the epigenetic regulation of gene transcription. Ascorbate uptake into most cells is through active transport by the sodium-dependent vitamin C transporter 2 (SVCT2). The aims of this study were to determine the kinetics of ascorbate uptake and retention by breast cancer cell lines under various oxygen conditions, and to investigate the role of SVCT2 in mediating ascorbate uptake and intracellular trafficking. Human MDA-MB231 cells accumulated up to 5.1 nmol ascorbate/10^6^ cells, human MCF7 cells 4.5 nmol/10^6^ cells, and murine EO771 cells 26.7 nmol/10^6^ cells. Intracellular ascorbate concentrations decreased rapidly after reaching maximum levels unless further ascorbate was supplied to the medium, and there was no difference in the rate of ascorbate loss under normoxia or hypoxia. SVCT2 was localised mainly to subcellular compartments, with the nucleus apparently containing the most SVCT2 protein, followed by the mitochondria. Much less SVCT2 staining was observed on the plasma membrane. Our data showed that careful management of the doses and incubation times with ascorbate in vitro allows for an approximation of in vivo conditions. The localisation of SVCT2 suggests that the distribution of ascorbate to intracellular compartments is closely aligned to the known function of ascorbate in supporting 2-OGDD enzymatic functions in the organelles and with supporting antioxidant protection in the mitochondria.

## 1. Introduction

Ascorbate, the most common form of vitamin C in physiological pH, is known for its potency as a reducing agent. Primates, including humans, are unable to synthesise vitamin C due to a functional loss of L-gulonolactone oxidase (GULO) [1]. Therefore, obtaining ascorbate from the diet and maintaining its cellular availability is necessary to support the many biological processes that require ascorbate. Under physiological conditions, cells are exposed to micromolar levels of circulating ascorbate in the blood stream, allowing its uptake through active transport by sodium-dependent vitamin C transporter 2 (SVCT2). Cells are able to accumulate and maintain millimolar ascorbate levels against the concentration gradient. Ascorbate levels vary between tissues, with the brain, adrenal glands, and liver among the organs accumulating the highest concentrations of up to 10 mM [2,3]. This suggests that there are different requirements for ascorbate across the different tissues, for specific molecular processes.

Other than acting as a potent antioxidant, ascorbate also demonstrates anti-cancer potential that may be dependent on its capacity to support the activity of enzymes, such as the hypoxia inducible factor (HIF)-hydroxylases and the epigenetic DNA and histone demethylases [4,5]. These enzymes belong to the 2-oxoglutarate dependent dioxygenase (2-OGDD) superfamily of enzymes that require ascorbate as a co-factor. When these enzymes are inactivated, their targets contribute to cancer cell growth and survival [6]. Support for these enzyme activities is dependent on the maintenance of intracellular ascorbate levels. However, the uptake and retention of ascorbate by cancer cells is not well understood.

Modelling ascorbate uptake by cells in vitro can be complicated. Ascorbate is unstable in a cell culture medium, and this instability could affect cell viability and the kinetics of ascorbate uptake. A cell culture medium contains trace amounts of iron which can catalyse the metal-dependent oxidation of ascorbate, resulting in the formation of dehydroascorbate (DHA), the oxidised form of ascorbate, and H_2_O_2_ [7,8,9]. Furthermore, ascorbate can react with molecular oxygen, also forming H_2_O_2_. These reactions contribute to the rapid loss of ascorbate from a cell culture medium, as well as potentially leading to cytotoxicity via H_2_O_2_ generation [8]. Therefore, it is important to assess the ideal conditions for ascorbate supplementation to promote the optimum cellular uptake, while preventing cytotoxicity.

Two isoforms of SVCT, SVCT1 and SVCT2, each have distinct functions in ascorbate transport. SVCT1 is distributed mainly in the epithelial cells of the intestines, and the kidneys, and is responsible for the bulk transport of ascorbate and the maintenance of ascorbate homeostasis. The distribution of SVCT1 supports its function for ascorbate uptake from the digestive system and ascorbate reabsorption in the kidney. SVCT1-mediated ascorbate transport is high capacity but low affinity (Km value for ascorbate range between 75–250 µM) [10]. On the other hand, SVCT2 is important for transporting ascorbate into every cell in the body, and thus displays a much broader distribution pattern [11]. In the epithelial cells of the human intestine, SVCT1 is expressed in the apical membrane while SVCT2 is found in the basolateral membrane [12]. A number of studies have reported a localisation of SVCT2 to various intracellular organelles but the reason for this remains largely unknown [5,13,14,15,16,17,18]. SVCT2 demonstrates low capacity but high affinity transport and the reported Km is in the range of 22–69 µM [10].

The first aim of this study was to investigate the pharmacokinetics of ascorbate uptake and retention in three breast cancer cell lines in vitro, in order to determine the optimal conditions for studying the ascorbate-dependent process in these cell lines. The effect of oxygen availability on kinetics was assessed, providing an insight into the stability of ascorbate in breast cancer cells. The second aim was to investigate the kinetics of SVCT2 localisation in different subcellular compartments in a human breast cancer cell line during ascorbate exposure. This enabled a better understanding of the relationship between intracellular SVCT2 shuttling and cellular ascorbate content.

## 2. Materials and Methods

*Materials*: Unless stated otherwise, all chemicals were from Sigma-Aldrich (St Louis, MO, USA).

*Cell culture*: Human breast cancer cell lines MDA-MB231 (negative for receptors for oestrogen, progesterone, and human epidermal growth 2) [19] and MCF-7 (positive for receptors for oestrogen, androgen, progesterone, and glucocorticoid) [20] were both from American Type Culture Collection (ATCC; Manassas, VA, USA), and mouse breast cancer cell line EO771 (a medullary breast cancer cell line derived from a spontaneous tumour in a C57BL/6 mouse) [21,22] was gifted by Dr Andreas Moeller (QIMR Berghofer Medical Research Institute, Herston, Australia.). All three cell lines were grown in Dulbecco’s Modified Eagle medium (DMEM, Gibco-BRL, Auckland, New Zealand) supplemented with 10% foetal bovine serum (Gibco-BRL), and 1% penicillin/streptomycin and tested to be mycoplasma negative. The cells were grown in humidified air at 37 °C with 5% CO_2_. Experiments under hypoxia were carried out in a Whitley H53 Hypoxistation (Don Whitley Scientific Limited, Shipley, UK) with N_2_/O_2_ mix at 37 °C with 5% CO_2_. Cell counts were carried out using an Improved Neubauer haemocytometer. Cell viability was assessed using 0.4% Trypan blue solution. The Trypan blue solution was added in a 1:1 dilution to the cell suspension, and the dilution was factored in when counting cells. Viable cells were represented as a percentage of the total cell number.

*Ascorbate uptake and retention*: Sodium ascorbate stock solution (100 mM) was freshly prepared and added to a culture medium to achieve final concentrations of 50–1000 µM. The cells were incubated with ascorbate for 2, 4, 6, 8 or 16 h before harvesting to analyse ascorbate uptake. Intracellular ascorbate retention was observed in normoxic and hypoxic conditions, with ascorbate retention under hypoxia investigated in the presence and absence of ascorbate in the medium. Cells were incubated with ascorbate in the cell culture medium to promote maximum uptake, then transferred to the Hypoxistation for further incubation for 2, 5, or 18 h. Ascorbate retention in the absence of ascorbate was done similarly, with ascorbate being removed (through medium replacement) before further incubation in the Hypoxistation for up to 24 h.

Cell culture medium was removed for ascorbate analysis and cells were harvested by trypsinization. The cells were washed and resuspended in phosphate buffered saline (PBS), and intracellular ascorbate was extracted and stabilised by adding ice cold 0.54 M perchloric acid (PCA) containing a metal chelator (diethylenetriamine pentaacetic acid, DTPA, 50 mM).

*Ascorbate measurement*: Ascorbate was measured using an Ultimate 3000 High Performance Liquid Chromatography (HPLC) system in reversed separation mode, coupled with a coulometric electrochemical detector (HPLC-ECD) (Thermo Fisher Scientific, Waltham, MA, USA), as described before [23]. Chromeleon 7 software (Dionex/Thermo Fisher Scientific Sunnyvale, CA, USA) was used for data acquisition and analysis of the peak area. A sodium L-ascorbate standard curve (40–1.25 µM) was freshly prepared in 77 mM PCA with 25 µM DTPA for each HPLC run. The stock ascorbate concentration was verified by spectrophotometry using the molar absorption coefficient for ascorbate 9860 M^−1^·cm^−1^ at 245 nm. The standard curve was plotted as a concentration (µM) of ascorbate with intracellular ascorbate calculated per million cells.

*Immunofluorescence staining*: MCF-7 cells were grown on glass coverslips in cell culture plates. The cells were supplemented with 500 µM of sodium ascorbate for up to 8 h under normoxia. Following incubation, the coverslips were fixed with a solution of acetone:methanol (1:1 *v*/*v*) at 4 °C, then washed with PBS, followed by permeabilization with 0.5% TritonX-100 in PBS. After a further wash, the cells were blocked with 1% BSA in PBS. The cells were labelled with antibodies against SVCT2 (HPA052825; 1:50) for ascorbate transporter localisation, against E-cadherin (ab76055; 1:100) as a cell membrane marker, and against HSP60 (sc-271215; 1:50) as a mitochondrial marker. Appropriate secondary antibodies containing fluorescent tags were employed to visualise the cells. Finally, coverslips were mounted onto glass slides using Prolong Diamond Antifade Mountant with DAPI (Invitrogen P36966, Auckland, NZ, USA) for imaging. The fluorescent images were taken using an Axio Imager (Carl Zeiss, Oberkochen, Germany) with 40× magnification. Each image was manually quantified for the number of cells (according to DAPI staining) and co-localisation of SVCT2 with subcellular markers assessed, presented as a percentage of the total cells in the field of view (*n* > 100).

*Statistics*: Results were plotted and statistically analysed using GraphPad Prism version 9.3.1. All experiments were done in triplicate and shown as mean ± SD. Statistical significance between ascorbate-untreated and ascorbate-treated was assessed with am unpaired *t*-test or a Dunnett’s one-way ANOVA with significance set at *p* < 0.05.

## 3. Results

### 3.1. Ascorbate Uptake Kinetics

**Figure 1:**
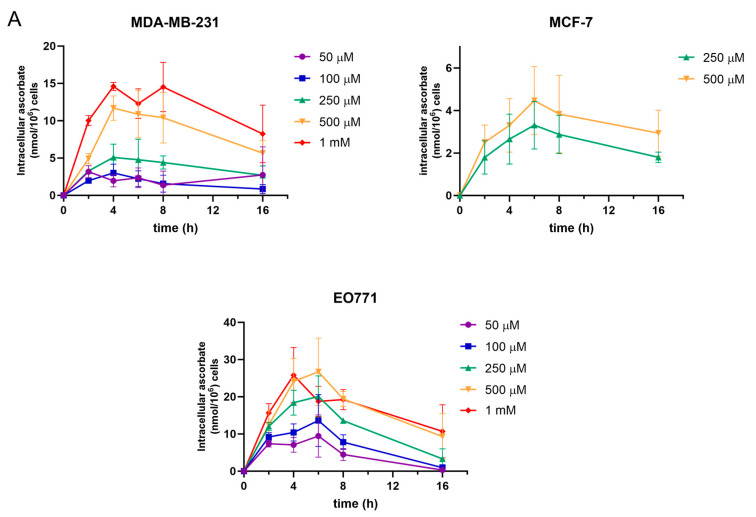

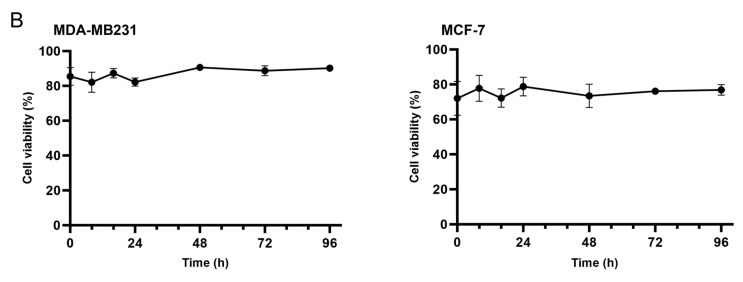
The effect of time and dose on ascorbate uptake by breast cancer cells. (**A**) MDA-MB231, MCF-7, and EO771 cells were exposed to 0–1000 µM for up to 24 h. Intracellular ascorbate concentrations, determined by HPLC-ECD, showed time-dependent ascorbate uptake with a maximum accumulation after 4–6 h, followed by a reduction over 16 h (mean ± SD; *n* = 3). (**B**) Selective doses of 250 µM (MDA-MB231) and 500 µM (MCF-7), and below, were shown to be non-toxic to cells as determined by cell viability measured with Trypan blue solution (mean ± SD; *n* = 3).

The cell lines used in this study were chosen to represent triple negative (MDA-MB231) or receptor positive (MCF-7) human breast cancers, with the addition of a mouse tumour cell line (EO771) commonly used in in vitro studies and in syngeneic tumour models [21,22]. These cell lines therefore represent two main human breast cancer models as well as a medullary breast cancer cell line, albeit of animal origin. The ascorbate accumulated in these human and murine breast cancer cells in a dose- and time-dependent manner, with the highest levels achieved when the cells were incubated with 500–1000 μM ascorbate (Figure 1). Maximal ascorbate accumulation occurred after 4–6 h of ascorbate supplementation in all three cell lines regardless of dose, and then intracellular concentrations reduced thereafter (Figure 1A). However, concentrations of 500 μM ascorbate and above proved to be cytotoxic to MDA-MB231, and 1 mM levels were cytotoxic to MCF7 and EO771 cells, judged by the changing of cell morphology and the loss of adherence. Ascorbate-mediated cytotoxicity due to the generation of H_2_O_2_ in tissue culture conditions is a well-documented phenomenon, and this toxicity becomes significant at concentrations around 1 mM [24,25,26,27]. Hence, higher ascorbate concentrations in cell culture should be avoided. We noted that the threshold for cytotoxicity differed between cell lines, and therefore ascorbate concentrations were limited to 250 μM for MDA-MB231 and 500 μM for MCF-7 and EO771 cells. Cell viability, assessed by morphology changes and trypan blue exclusion, was not affected by these doses following ascorbate supplementation for 24 h (Figure 1B). MDA-MB231 cells exposed to 250 μM ascorbate accumulated 5.1 ± 1.4 nmol ascorbate/10^6^ cells after 4 h, and MCF7 and EO771 cells with 500 μM ascorbate contained 4.5 ± 1.3 nmol/10^6^ cells and 26.7 ± 7.4 nmol/10^6^ cells after 6 h, respectively.

The ascorbate uptake varied between the cell lines. The cancer cells also exhibited a rapid decline in ascorbate content after reaching maximum intracellular levels (Figure 1A). By 16 h of supplementation, the intracellular ascorbate had reduced to similar or lower concentrations than those measured at 2 h. This was particularly noticeable for the EO771 cells, with a 2–5-fold reduction at 16 h compared to the maximum levels at 6 h.

**Figure 2:**
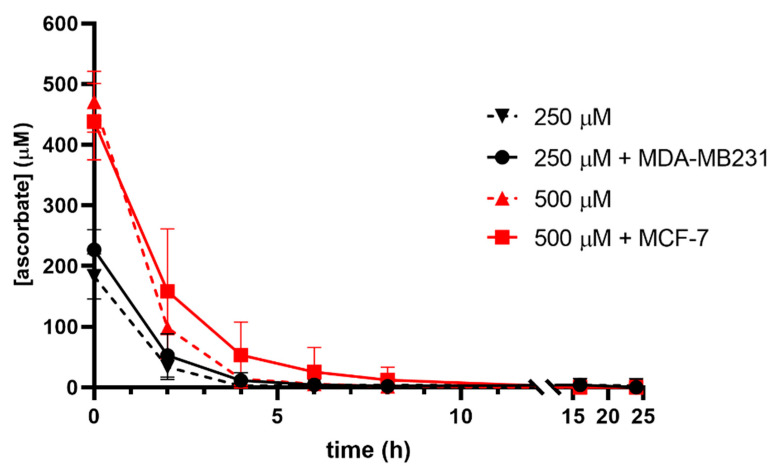
Ascorbate stability in culture medium in the absence and presence of cells. Cell culture medium DMEM, supplemented with 250 μM or 500 μM ascorbate, with and without cells, was sampled over 24 h and ascorbate measured by HPLC-ECD. MDA-MB231 cells were incubated with 250 μM ascorbate and MCF-7 with 500 μM ascorbate. Ascorbate degraded rapidly in the medium within 4 h of supplementation, and the presence of cells did not affect this loss (mean ± SD; *n* = 3).

As ascorbate is readily oxidised in vitro, the stability of ascorbate in a cell culture medium was assessed with and without cells to determine whether the drop in the intracellular ascorbate was due to the loss of ascorbate from the medium (Figure 2). The expected ascorbate concentrations were measured in the cell culture medium supplemented with 250 µM and 500 µM immediately after addition but decreased rapidly over the first 4 h under normoxia. The presence of cells had only a minimal effect on this loss from the medium, despite the ascorbate being taken up by cells. No ascorbate was detected in the culture medium after overnight incubation, either with or without cells present.

**Figure 3:**
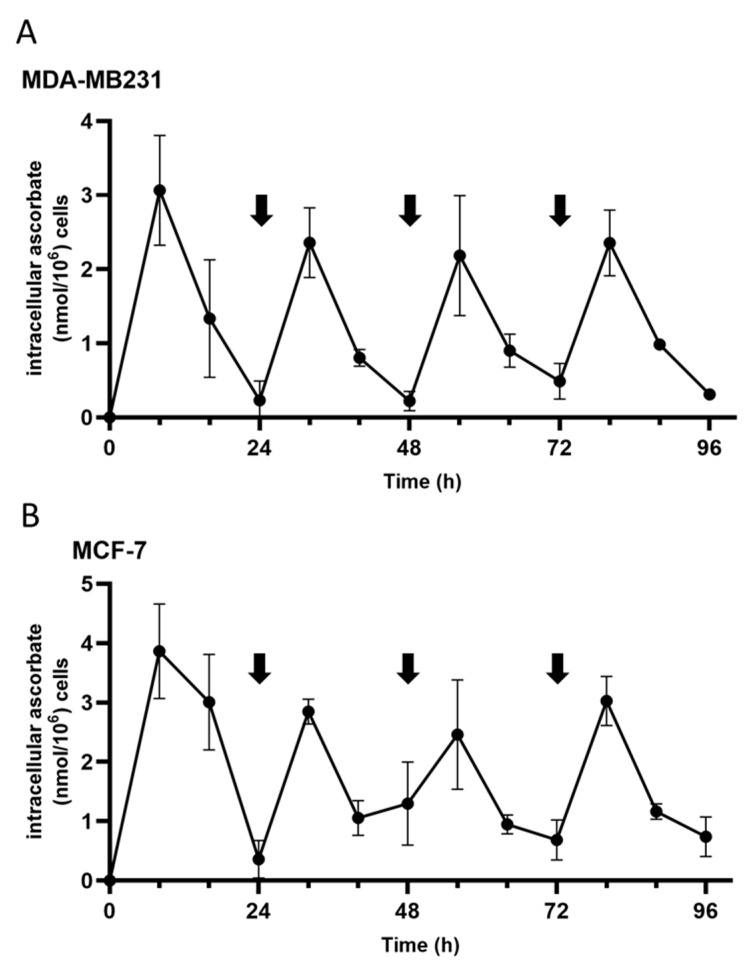
Intracellular ascorbate levels in MDA-MB321 and MCF-7 cells upon repeated supplementation of ascorbate. (**A**) MDA-MB231, and (**B**) MCF-7 cells were supplemented with 250 µM and 500 µM, respectively, for up to 96 h. Ascorbate was refreshed every 24 h (vertical arrows) to the fresh cell culture medium, along with 20 µg/mL of catalase to prevent H_2_O_2_-induced cytotoxicity. The intracellular ascorbate was measured every 8 h. Ascorbate supplementation increased intracellular ascorbate levels before depleting within 24 h (mean ± SD; *n* = 3).

Due to this rapid turnover of ascorbate in both the cells and the cell culture medium, we tested the effect of repeated supplementation on the intracellular ascorbate levels (repeated supplementation shown by black arrows in Figure 3). To avoid the potential cytotoxic effect due to accumulated H_2_O_2_, catalase (20 µg/L) was added with the ascorbate. Repeated supplementation showed spikes in the intracellular ascorbate levels at 8 h following the ascorbate addition before a rapid reduction to baseline levels at 24 h post supplementation in both MDA-MB231 and MCF-7 cell lines (Figure 3). Interestingly, the highest ascorbate levels were reached after the first supplementation, with lower peak levels at the subsequent supplementations.

**Figure 4:**
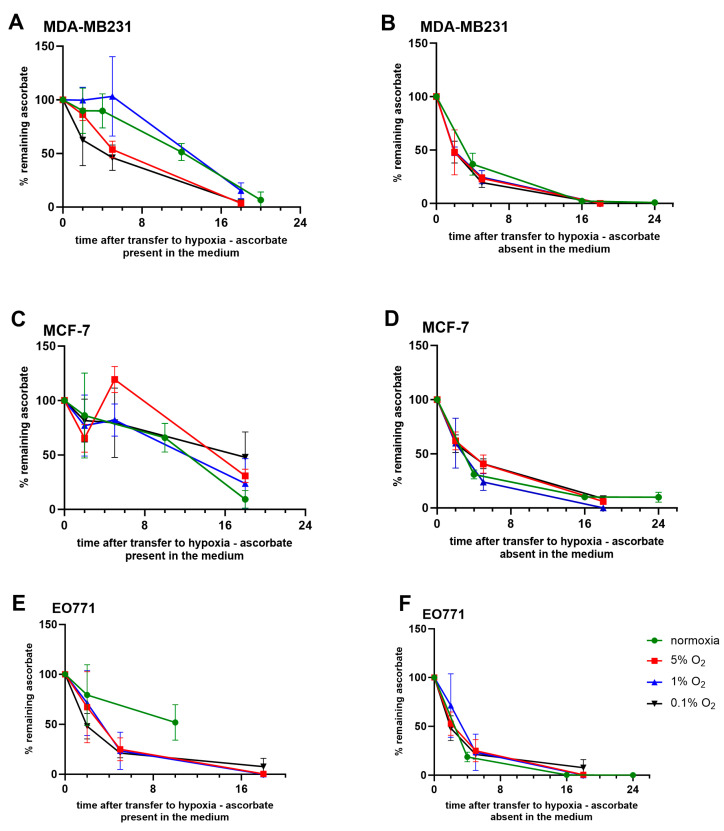
Ascorbate retention in MDA-MB231, MCF-7, and EO771 under decreasing oxygen conditions, with or without ascorbate removal from the cell culture medium. Cells were supplemented with 250 μM for 4 h (MDA-MB231) and 500 μM for 6 h (MCF-7 and EO771) before exposure to decreased oxygen conditions. Upon re-incubation at these times, (**A**,**C**,**E**) ascorbate was either maintained in the medium, or (**B**,**D**,**F**) the medium was replaced with fresh medium not containing ascorbate. Intracellular ascorbate was measured by HPLC-ECD for up to 18 h and shown as a percentage of the maximum ascorbate levels for each cell line. Normoxia (green), 5% O_2_ (red), 1% O_2_ (blue) and 0.1% O_2_ (black); (mean ± SD; *n* = 3).

To assess whether oxygenation conditions affected the ascorbate turnover or retention, MDA-MB231, MCF-7, and EO771 cells were supplemented with ascorbate to achieve optimum uptake under normoxia (250 µM for 4 h, or 500 µM for 4–6 h, respectively), then incubated under various oxygen conditions (normoxia to 0.1% O_2_) without replacing the medium (Figure 4A,C,E). The intracellular ascorbate levels in MDA-MB231 and MCF-7 cells decreased over time regardless of oxygenation (Figure 4A,C). EO771 cells appeared to exhibit a more rapid ascorbate loss when incubated under hypoxic conditions compared to normoxic incubation compared to the human cells (Figure 4E).

To determine if the retention of intracellular ascorbate changed when the ascorbate was withdrawn following optimal ascorbate loading, the experiment was repeated but any remaining extracellular ascorbate was removed before hypoxic incubation (Figure 4B,D,F). Again, the intracellular ascorbate levels declined rapidly within hours of ascorbate removal. The declines in the intracellular ascorbate were observed in all oxygen conditions, and appeared to be more rapid when the ascorbate was withdrawn compared to when the ascorbate was left in the media (Figure 4A,C,E vs. Figure 4B,D,F). At the time of cell transfer to the Hypoxistation (4–6 h after the original ascorbate addition), there was very little ascorbate in the medium (Figure 2). The extended retention of the intracellular ascorbate may be due to the remaining ascorbate in the medium being protected from oxidation in the hypoxic environment [28].

### 3.2. SVCT2 Localisation and Kinetics

The transport of ascorbate from the extracellular milieu into the cells is mediated by SVCT2. To better understand the dynamics of ascorbate uptake, the localisation of SVCT2 during ascorbate exposure was assessed by immunofluorescence in MCF-7 cells. The presence of SVCT2 was monitored every 2 h during ascorbate supplementation.

**Figure 5:**
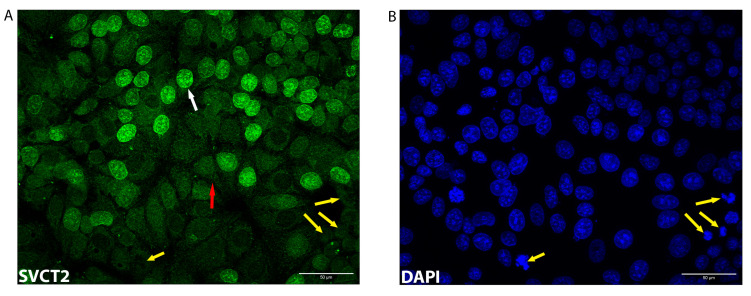
A representative image of SVCT2 localisation in MCF-7 cells. MCF-7 cells were exposed to ascorbate and labelled for (**A**) SVCT2 in green, and (**B**) DNA in blue (2 h supplementation, scale bar = 50 μm). SVCT2 staining shows mainly intracellular localisation with emphasis around nuclei (white arrow) although not all nuclei were stained positive. Potential association between cell mitosis and the negative nuclear SVCT2 localisation shown by yellow arrows. Faint staining of SVCT2 was observed on the cellular membrane (red arrow).

As shown in Figure 5A, SVCT2 staining was detected intracellularly with staining throughout the cytoplasm and distinct staining around the nuclei (white arrow, Figure 5A). However, not all nuclei were positive for SVCT2 (Figure 5). The reason for this variation is unclear. Some of the lack of staining may reflect the transient changes in the localisation of SVCT2 during the different phases of the cell cycle. The yellow arrows show examples of the cells undergoing cell division, marked by the condensed chromosomes (Figure 5A,B) that did not show nuclear SVCT2 staining. The dissolution of the nuclear membrane during cell mitosis [29] might be linked to the negative staining of SVCT2. 

**Figure 6:**
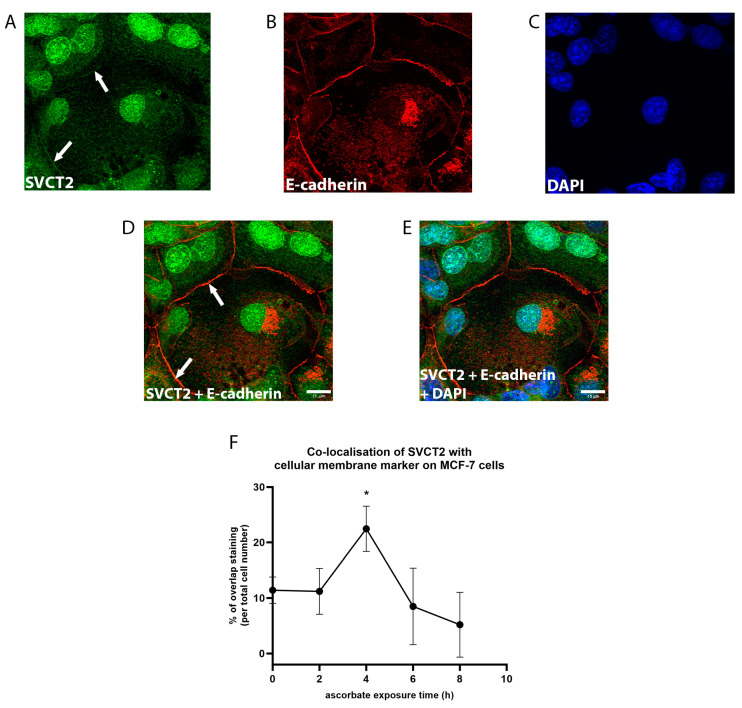
SVCT2 localisation on the cell membrane in MCF-7 cells. MCF-7 cells were exposed to ascorbate and labelled for (**A**) SVCT2 in green, (**B**) E-cadherin in red, and (**C**) DAPI in blue (representative images at 4 h). The overlap between SVCT2 and E-cadherin staining (white arrows; (**A**,**D**)) represented the (**D**) co-localisation of the transporter with E-cadherin, and (**E**) DAPI. Scale bar = 15 μm (**F**) The number of cells with SVCT2-E-cadherin overlap were manually counted and compared during ascorbate supplementation over 8 h; * unpaired *t*-test (0 h vs. 4 h, *p* = 0.01; mean ± SD; *n* = 3).

SVCT2 staining was much less apparent at the cell membrane. Faint staining was detected (red arrow, Figure 5A), and localisation at the cell membrane was confirmed by co-staining with E-cadherin [30] (Figure 6). The strongest staining at the cell membrane was detected following 4 h of ascorbate supplementation (Figure 6F), with levels at this time point being significantly higher than at the other times (*p* = 0.01). Interestingly, SVCT2 membrane staining declined at later time points with the lowest detection at 8 h following the addition of ascorbate (Figure 6F).

**Figure 7:**
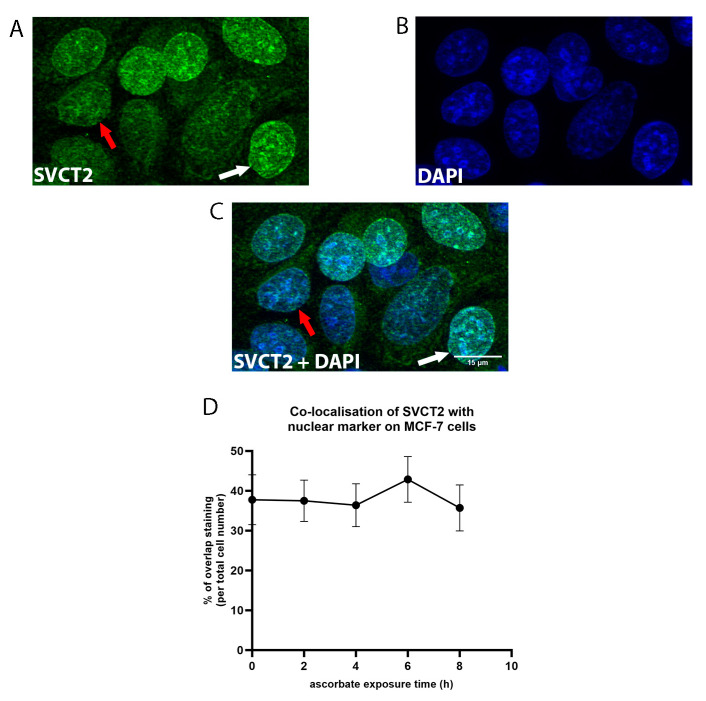
SVCT2 localisation on the nucleus of MCF-7 cells. MCF-7 cells were exposed to ascorbate and labelled with (**A**) SVCT2 in green, and (**B**) DAPI in blue (representative images at 2 h, magnification = 40×; scale bar = 15 μm). (**D**) The number of cells with overlap between SVCT2 (white arrow represents staining within nucleus, red arrow represents staining on nuclear membrane) and DAPI (**C**) were manually counted and compared at each time point during ascorbate supplementation; one-way ANOVA (mean ± SD; *n* = 3).

SVCT2 staining strongly overlapped with DAPI staining, indicating nuclear localisation of the transporter, with over one third of the cells in the field showing this staining pattern (Figure 5 and Figure 7). The number of cells that exhibited SVCT2 nuclear localisation persisted throughout ascorbate supplementation, with no significant difference over time (Figure 6D, one-way ANOVA, *p* = 0.7). Staining within the nucleus (white arrow; Figure 7A,D) as well as the nuclear membrane (red arrow; Figure 7A,D) was evident.

**Figure 8:**
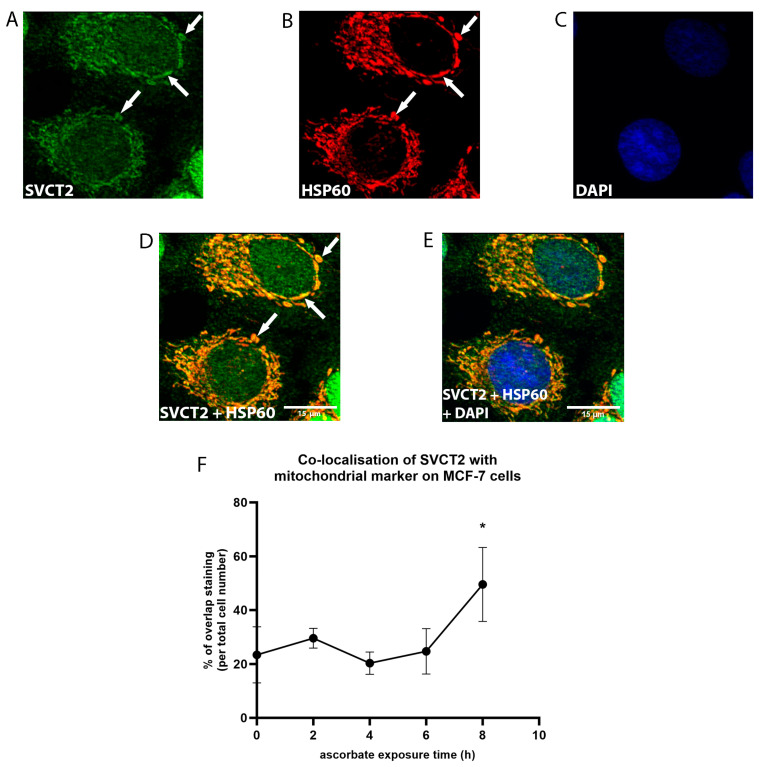
SVCT2 localisation on the mitochondria in MCF-7 cells. MCF-7 cells were exposed to ascorbate and labelled with (**A**) SVCT2 in green, (**B**) mitochondrial HSP60 in red, and (**C**) DAPI in blue (representative images at 8 h, magnification = 40×). (**D**) The mitochondrial co-localisation of SVCT2 shown in the merge images of SVCT2 and HSP60 (white arrows represent the overlap between SVCT2 and HSP60 staining) and (**E**) merged with DAPI. Scale bar = 15 μm (**F**) The number of cells with SVCT2 co-localised with HSP60 were manually counted and compared during ascorbate supplementation over 8 h. * The mitochondrial co-localisation of SVCT2 was detected in the highest proportion of cells at 8 h of ascorbate exposure and is significant compared to the basal level (one-way ANOVA; *p* = 0.01; mean ± SD; *n* = 3).

SVCT2 staining was also observed in the cytosol, where it appeared to be localised to organelles. We used HSP60 as a marker for mitochondria [31] to investigate the localisation of SVCT2 to the mitochondria (Figure 8). The white arrows indicate the overlapped staining between SVCT2 (Figure 8A) and HSP60 (Figure 8B). The data showed that mitochondrial SVCT2 staining increased from ~20–30% of the cells in the first 6 h to ~50% by 8 h of ascorbate supplementation (Figure 8F, one-way ANOVA, *p* = 0.01). SVCT2 staining not overlapping with HSP60 was also observed in the cytosol, indicating the potential localisation of SVCT2 on other organelles.

## 4. Discussion

Our study has shown that ascorbate was accumulated rapidly by breast cancer cells in vitro but was lost after the optimum intracellular levels were reached, regardless of ascorbate presence, oxygenation, and SVCT2 cellular localisation. The ascorbate concentrations that were added to the tissue culture medium were carefully managed to avoid H_2_O_2_-induced cytotoxicity [24,25,26,27]. We noted that the ascorbate concentrations tolerated by the cell lines differed, with MDA-MB231 cells being more vulnerable to ascorbate-induced toxicity. These results demonstrate the caution that is needed when using ascorbate in tissue culture conditions.

Cells in culture are generally deficient in ascorbate as most cell culture media do not contain any ascorbate. A lack of ascorbate can affect their functions, and maintaining the levels of intracellular ascorbate is essential to support the activity of ascorbate-requiring enzymes such as the 2-OGDDs. These enzymes are involved in many biological processes, for instance in collagen biosynthesis, fatty acid metabolism, hormone synthesis, the hypoxic response, and epigenetic regulation [6]. Adequate intracellular ascorbate levels were shown to modulate the hypoxic response and epigenetic regulation through members of 2-OGDDs, the HIF-hydroxylases [32], and ten-eleven translocases (TETs) [14], respectively. The ascorbate supplementation in various types of cancer cells [32,33] and tumour-bearing mice [34] limited HIF-pathway activation, which may contribute to decreased tumour survival and progression. Similarly, the ascorbate supplementation in acute myeloid leukaemia cells in vitro contributed to the restoration of TET activities, leading to the increased ratio of 5-hydroxymethyl cytosine to 5-methyl cytosine [14,35], an epigenetic marker linked to reversal of transcriptional silencing. 

The ascorbate uptake in the three breast cancer cell lines in our study was rapid, achieving maximal intracellular levels within 4–6 h for the human breast cancer cells. Both rate and extent of the uptake compared closely with data reported for human renal carcinoma, with similar intracellular levels of 5.4–8.9 nmol ascorbate/10^6^ cells [36], colon and endometrioid carcinoma cells, and T cell leukaemia cells, which accumulated 2–4 nmol ascorbate/10^6^ cells after 16 h of supplementation [32]. The murine breast cancer cell line EO771 accumulated a significantly higher ascorbate concentration within 6 h. There is limited literature available on the ascorbate content of mouse cancer cells, but the levels in EO771 cells were much higher than we previously found in other mouse cancer cells, estimated at 1–5 nmol/10^6^ cells in LL2 lung cancer cells, B16-F10 melanoma, and CMT-93 colorectal cancer cells [34,37]. The reason for the high ascorbate levels in EO771 breast cancer cells, and the rapid turnover, is unknown, but possibly reflects a higher requirement for the vitamin in these fast-growing cells. The rapid accumulation in all three breast cancer cell lines suggests a high need for ascorbate in breast cancer cells.

Based on the intracellular water volume of cancer cells determined in Ishikawa and WiDr cells [32] and Caco-2 cells [38], the average water volume in cancer cells is ~2.6 µL/10^6^ cells. Assuming a similar volume for MDA-MB231 and MCF-7 cells, the intracellular ascorbate concentrations achieved by ascorbate supplementation were calculated to be ~1.9 mM and ~1.7 mM, respectively, which is equivalent to the intracellular levels found in vivo [3,39]. The levels of ascorbate accumulated by EO771 (26.7 nmol/10^6^ cells) is notably higher, calculated to be equivalent to ~10 mM.

The SVCTs are the main transport system that require two sodium ions to transport one molecule of L-ascorbic acid. An alternative mechanism of uptake includes a facilitated diffusion of ascorbic acid, the predominant form under acidic conditions. DHA is taken up by glucose transporters [3] and subsequently reduced to ascorbate inside the cell. Despite the competition from glucose [40], this mode of transport has been observed in neutrophils, and erythrocytes that are glycolytic and express high levels of the GLUTs [41,42]. DHA is unstable at neutral pH and very little is present in plasma or extracellular tissue [8]. It is therefore considered that the active transport of ascorbate via SVCTs is the predominant mechanism of uptake.

Given the role of SVCT2 as the main transporter of ascorbate to the cells, it was expected that SVCT2 is widely expressed on the cell membrane to mediate the ascorbate accumulation. Surprisingly, the SVCT2 staining on the membrane was detectable only as a weak signal. This agrees with previous data in renal cell carcinoma cells, where the SVCT2 protein was not detected on the cell membrane [36]. Although the SVCT2 content of the cell may not change, immunolocalisation suggests that there are transient changes in cellular localisation of the transporter. These differences will not be detected by western blotting, but our studies have indicated that there is trafficking of the SVCT2 from the plasma membrane and the mitochondria, whereas staining of the nucleus was more stable. Nevertheless, the proportion of cells with membrane-localised SVCT2 increased at 4 h of ascorbate exposure. This potentially mediated maximum uptake as the highest intracellular ascorbate levels were detected at 6 h of ascorbate supplementation.

SVCT2 staining was stronger in the nucleus and nuclear membrane. Because ascorbate is known as a co-factor of 2-OGDD enzymes, such as TETs and KDMs [4,5,43], we speculated that ascorbate is transported to the nucleus specifically to support the functions of these enzymes. Our results, that indicate that about 1/3 of the cells showed nuclear SVCT2 localisation prior to ascorbate exposure, suggest that ascorbate distribution to the nucleus is a high priority for the cells. This localisation may be particularly important for cancer cells because aberrant DNA and histone methylation have been associated with tumorigenesis and malignancies [6,44]. 

SVCT2 staining was also evident in the intracellular compartments, indicating the importance of ascorbate distribution to the organelles for functional purposes. An analysis of the time of SVCT2 distribution to the mitochondria indicates that SVCT2 mediates the ascorbate transport from the cytosolic compartment into the mitochondria when, or soon after, the cells reach their maximum cellular accumulation. The mitochondrial SVCT2 distribution could reflect the importance of ascorbate to neutralise oxidants generated from the reactions taking place in the mitochondria, such as in the electron transport chain [45,46]. The increase in mitochondrial SVCT2 localisation after 8 h of ascorbate supplementation could reflect a potential response to H_2_O_2_-mediated cytotoxicity as a result of the oxidation of ascorbate in the cell culture medium.

SVCT2 signal was also detected in the cytoplasm as spots that did not correspond to HSP60 staining, indicating potential localisation of SVCT2 on other organelles, such as the lysosomes, Golgi, and the endoplasmic reticulum. The potential shuttling of SVCT2 between intracellular compartments during ascorbate exposure, shown here in breast cancer cells, is a new and important finding because it provides new information on the distribution of ascorbate inside the cells over time, and potentially supports the anti-cancer function of ascorbate [47].

The rapid uptake of ascorbate by the breast cancer cells was followed by rapid ascorbate loss, with intracellular ascorbate levels reducing to baseline within 24 h of supplementation. This has not been reported for other cell types [32,36,48] and may be peculiar to breast cancer cells. There are several possible reasons for this high turnover. Breast cancer cells, specifically, may have an increased consumption of ascorbate due to the high oxidative stress and the active 2-OGDD enzymes. Ascorbate is known to be consumed by the uncoupled reaction catalyzed by the 2-OGDDs [49], but the extent to which this occurs in breast cancer cells is unknown. Our data indicating high rates of turnover suggest that this may be significant. Turnover may also be caused by an imbalance between ascorbate oxidation and the reduction of DHA which leads to the hydrolysis and irreversible degradation of DHA [8]. DHA has a short half-life (approximately 6 min [8,50,51]) and the factors that can accelerate DHA degradation include insufficient levels of glutathione, NAPDH, and glutaredoxin that are required to reduce DHA. The levels of these antioxidants can vary substantially between cancer cells [52].

The hypoxic conditions did not seem to affect the rapid turnover of intracellular ascorbate suggesting that oxygen is not a limiting factor in ascorbate turnover in these cancer cells. Hypoxia may prolong the availability of ascorbate in a cell culture medium, as shown previously in ascorbate supplementation on HT29 cells [28]. This could be the contributing factor for ascorbate retention when some ascorbate was maintained in the cell culture medium during exposure to hypoxia [28] compared to ascorbate withdrawal.

We were able to replenish the intracellular ascorbate by repeated supplementation of ascorbate to the medium. We utilised catalase to prevent the accumulation of H_2_O_2_ as a result of the oxidation of accumulated ascorbate in the cell culture medium, for repeated ascorbate exposure. However, the constant monitoring of the culture medium conditions does pose a barrier to the routine supplementation of the tissue culture media, and this inconvenience results in the addition of ascorbate to the cells in vitro occurring only infrequently. We also noted that the cells differed in their susceptibility to ascorbate-induced cytotoxicity, with MDA-MB231 cells being more sensitive than the other cells. The reason for this difference is unknown but may be related to an underlying susceptibility of the MDA-MB231 cells to redox stress. Previous studies have indicated changes in mitochondrial redox stress [53] and in antioxidant capacity [54] in these cells. Despite this difference, MDA-MB231 cells accumulated a similar intracellular ascorbate concentration to MCF-7 cells.

Given the requirement of ascorbate to sustain the vital enzyme activities that control cell metabolism and gene expression, ensuring that the cultured cells contain sufficient ascorbate without inducing H_2_O_2_-mediated cytotoxicity is important. The kinetics of the uptake and rapid turnover of ascorbate in endothelial cells showed a similar profile to what we have observed in the breast cancer cells, with respect to the dependence on the repeated addition to the medium in order to maintain stable intracellular levels [48]. These authors showed that using 2-phosphoascorbate, a form of ascorbate not prone to autoxidation, resulted in more stable intracellular levels. The use of 2-phosphoascorbate may also help prevent the cytotoxicity effect of the H_2_O_2_ build up and the rapid ascorbate loss in the cell culture medium over time [48]. However, the kinetics of ascorbate uptake from 2-phosphoascorbate differs [14], as it is dependent on the initial release of ascorbate by cell surface phosphatases [55]. Therefore, the uptake of sodium ascorbate from 2-phosphoascorbate would need to be determined separately.

Overall, our findings highlight the challenges in sustaining cellular ascorbate levels over time in cultured cells and suggest the necessity for ascorbate resupplementation for experimental designs that require several days of incubation.

## 5. Conclusions

Ascorbate is rapidly accumulated and depleted by the breast cancer cells. A continuous supply of ascorbate in vitro is required to ensure the availability of ascorbate to these cells. Intracellular ascorbate loss occurred rapidly under both normoxia and hypoxia. The dominant presence of SVCT2 intracellularly in the nucleus and mitochondria indicates that ascorbate distribution to the subcellular compartments is important following the uptake into the cell from the extracellular milieu.

## Data Availability

All data generated or analysed during this study are included in this published article.
